# Phase 1 randomized controlled trial to evaluate the safety and immunogenicity of recombinant *Pichia pastoris*-expressed *Plasmodium falciparum* apical membrane antigen 1 (PfAMA1-FVO [25-545]) in healthy Malian adults in Bandiagara

**DOI:** 10.1186/s12936-016-1466-4

**Published:** 2016-08-30

**Authors:** Mahamadou A. Thera, Drissa Coulibaly, Abdoulaye K. Kone, Ando B. Guindo, Karim Traore, Abdourhamane H. Sall, Issa Diarra, Modibo Daou, Idrissa M. Traore, Youssouf Tolo, Mady Sissoko, Amadou Niangaly, Charles Arama, Mounirou Baby, Bourema Kouriba, Mahamadou S. Sissoko, Issaka Sagara, Ousmane B. Toure, Amagana Dolo, Dapa A. Diallo, Edmond Remarque, Roma Chilengi, Ramadhani Noor, Sanie Sesay, Alan Thomas, Clemens H. Kocken, Bart W. Faber, Egeruan Babatunde Imoukhuede, Odile Leroy, Ogobara K. Doumbo

**Affiliations:** 1 Malaria Research and Training Centre, Department of Epidemiology of Parasitic Diseases, Faculty of Medicine and Dentistry, University of Sciences, Techniques and Technologies, Bamako, Mali; 2 African Malaria Network Trust (AMANET), P.O. Box 33207, Dar Es Salaam, Tanzania; 3 Biomedical Primate Research Center (BPRC), P.O. Box 3306, 2280 GH Rijswijk, The Netherlands; 4 Center for Infectious Diseases Research in Zambia (CIDRZ), P.O. Box 34681, Lusaka, 10101 Zambia; 5 European Vaccine Initiative, European Vaccine Initiative, Im Neuenheimer Feld 307, 69120 Heidelberg, Germany; 6 Medical Research Council, P.O. Box 273, Banjul, The Gambia

**Keywords:** Malaria, Vaccine, Safety, Immunogenicity, Blood-stage, PfAMA1, *Plasmodium falciparum* antigen

## Abstract

**Background:**

The safety and immunogenicity of PfAMA1, adjuvanted with Alhydrogel^®^ was assessed in malaria–experienced Malian adults. The malaria vaccine, PfAMA1-FVO [25-545] is a recombinant protein *Pichia pastoris*-expressed AMA-1 from *Plasmodium falciparum* FVO clone adsorbed to Alhydrogel^®^, the control vaccine was tetanus toxoid produced from formaldehyde detoxified and purified tetanus toxin.

**Methods:**

A double blind randomized controlled phase 1 study enrolled and followed 40 healthy adults aged 18–55 years in Bandiagara, Mali, West Africa, a rural setting with intense seasonal transmission of *P. falciparum* malaria. Volunteers were randomized to receive either 50 µg of malaria vaccine or the control vaccine. Three doses of vaccine were given on Days 0, 28 and 56, and participants were followed for 1 year. Solicited symptoms were assessed for seven days and unsolicited symptoms for 28 days after each vaccination. Serious adverse events were assessed throughout the study. The titres of anti-AMA-1 antibodies were measured by ELISA and *P. falciparum* growth inhibition assays were performed.

**Results:**

Commonest local solicited adverse events were the injection site pain and swelling more frequent in the PfAMA1 group. No vaccine related serious adverse events were reported. A significant 3.5-fold increase of anti-AMA-1 IgG antibodies was observed in malaria vaccine recipients four weeks after the third immunization compared to the control group.

**Conclusion:**

The PfAMA1 showed a good safety profile. Most adverse events reported were of mild to moderate intensity. In addition, the vaccine induced a significant though short-lived increase in the anti-AMA1 IgG titres.

Registered on www.clinicaltrials.gov with the number NCT00431808

**Electronic supplementary material:**

The online version of this article (doi:10.1186/s12936-016-1466-4) contains supplementary material, which is available to authorized users.

## Background

Despite recent renewed malaria control efforts, the disease still caused in 2013, 438,000 deaths (uncertainty range 236,000–635,000), 90 % of which occurred in Africa south of the Sahara [1]. An effective malaria vaccine would be a valuable additional tool particularly if one considers the new impetus towards malaria elimination [2]. Apical membrane antigen-1 (AMA1) is a surface protein expressed during the asexual blood stage of *Plasmodium falciparum*, and a leading blood stage vaccine candidate, with different formulations assessed and being tested in malaria endemic areas in Africa [3–5]. Three AMA-1-based vaccines have been evaluated in clinical trials in Mali, including two different monovalent vaccines based on AMA-1 derived from the 3D7 and FVO clones of *P. falciparum,* respectively, [5] and a bivalent vaccine that includes both of these versions of AMA-1 [6]. Preclinical studies [7, 8] of AMA1-based vaccines as well as clinical trials in people not naturally exposed to malaria [9, 10] have shown promise.

The trial reported here used PfAMA1-FVO [25-545], a lyophilized preparation of the ectodomain of the FVO clone of *P. falciparum* AMA1 [11]. This first phase 1 trial in malaria-exposed adults was the logical sequence of the clinical development of PfAMA1-FVO [25-545], and took place after a phase 1a trial done in Nijmegen [9]. The aim was to assess the malaria vaccine candidate immunogenicity and reactogenicity in adults naturally exposed to malaria in Mali.

## Methods

This was a double blind conducted, randomized, controlled clinical trial designed to assess the safety and immunogenicity of the blood stage malaria vaccine candidate PfAMA1 [25-545] of *P. falciparum* FVO strain, adjuvanted with Alhydrogel^®^.

### Study setting

The study was conducted at the Bandiagara Malaria Project (BMP) research clinic located close to the district hospital in Bandiagara, a rural setting with 13,634 inhabitants in the Dogon country in northeast Mali. Mean annual rainfall is 600 mm and *Anopheles gambiae* is the principal malaria vector. Malaria transmission is highly seasonal and overlaps with the rainy season [12]. *Plasmodium falciparum* is the most frequent parasite species causing 97 % of malaria infections. Malaria burden is heavy, children aged less than 10 years have an average of two clinical malaria episodes every transmission season [12, 13] and severe malaria afflicts one in 50 children aged less than 6 years each year [14]. Older children and adults are relatively protected against malaria disease but remain susceptible to malaria infection.

### Participants

Participants were healthy adult men and women residing in Bandiagara aged 18–55 years old. After a screening for eligibility they were included if they planned to remain in Bandiagara for at least 12 months, showed good general health based on history, clinical and laboratory examinations and gave written informed consent. Female volunteers had in addition to declare willingness not to become pregnant during the first 5 months of the study, and they were referred to existing birth control programme at the district health care centre. Exclusion criteria were significant current illness as indicated by history, examination and/or laboratory testing including complete blood counts, alanine aminotransferase (ALT) and serum creatinine; previous immunization with any experimental vaccine; chronic use of immunosuppressants; receipt of blood products during the previous 6 months; and allergy to substances present in the vaccines.

### Ethical clearance

The Institutional Review Board of the University of Bamako Faculty of Medicine approved the protocol and the informed consent forms, approval letter 07-35/FMPOS dated on May 15, 2007. The informed consent forms specified that the trial data will be published and that the participants confidentiality will be preserved by using only anonymous study numbers and no reference to particular individual identity. After obtaining community permission as described [15], the trial was publicized by local radio broadcast. The trial was conducted in compliance with the International Conference on Harmonisation Good Clinical Practices, the Declaration of Helsinki and applicable regulatory requirements of Mali. Separate written informed consent was obtained for screening and enrolment. Verbal consent of illiterate participants was administered and then documented using their fingerprints, a process verified by signatures of independent witnesses. Permission to import the investigational products in Mali was given by the Republic of Mali Ministry of Health. The trial sponsor was the African Malaria Network Trust (AMANET). A safety monitoring committee set by AMANET reviewed the safety data after the first and second vaccine dose and gave authorization to administer respectively the second and third vaccine doses.

### Interventions and participant surveillance

PfAMA1 antigen comprises amino acids 25-545 corresponding to the ectodomain of AMA1 derived from the FVO clone of *P. falciparum*. Vaccine production and formulation are described elsewhere [11]. The vaccine was manufactured under GMP conditions by Eurogentec SA, Belgium, and supplied as lyophilized single dose vials containing 62.5 µg AMA1 protein, 23.3 µg EDTA, 25 mg saccharose, 187 µg NaH_2_PO_4_·2H_2_O, 226 µg Na_2_HPO_4_. The adjuvant used, Alhydrogel^®^ is a crystalline aluminium oxyhydroxide AlOOH, also known as boehmite supplied as a 0.2 % suspension by Staten Serum Institute (SSI), Denmark. Vials were reconstituted by adding 625 µL 0.2 % Alhydrogel^®^ suspension; the adsorption to Alhydrogel^®^ was previously confirmed to be greater than 99 %. The reconstituted vaccine was then incubated for 60 min at room temperature to facilitate adsorption to the Alhydrogel^®^ and a dose of 0.5 mL containing 50 µg AMA1 and approximately 0.5 mg aluminium was used for injection.

The control vaccine, tetanus toxoid was also manufactured at SSI. The tetanus toxoid was produced from a formaldehyde detoxified and purified tetanus toxin. The vaccine was supplied in pre-filled syringes of 0.5 mL containing more than 20 IU tetanus toxoid, aluminum hydroxyde as adjuvant and thiomersal as preservative. A buffer solution, water for injection, was added. All doses of all vaccines were administered by intramuscular injection in the left deltoid muscle.

The study vaccines were given on study days 0, 28 and 56, the first vaccine dose being injected in May 2007. Study day 84 clinic visit was done in August, at the peak of malaria transmission. Study day 140 clinic visit took place at the height of the dry season. The final study follow-up on day 365 coincided with the beginning of the 2008 malaria season. Interim safety reports were reviewed by an independent safety monitoring committee before the second and third vaccine doses. Active surveillance of participants for 28 days after each immunization was completed in August 2008 corresponding to study day 84. The database was locked for the primary unblinded analysis after study day 84, and the study continued in a single-blinded fashion, although individual study allocations were not disclosed to on-site study investigators or staff with the exception of the principal investigator. The extended surveillance phase included continuous access to free basic medical care at the research clinic, monthly home visits, and scheduled visits on study days 140 and 364. All participants completed the follow-up schedule and were included in the analysis.

### Outcomes

The primary outcome was safety, measured through the occurrence of solicited symptoms during a 7-day follow-up period after immunization (day of immunization and days 1, 3 and 7 after immunization); the occurrence of unsolicited symptoms during a 28-day follow-up period after immunization (day of immunization and 28 subsequent days); the occurrence of laboratory toxicities; and the occurrence of serious adverse events during the entire study period. Secondary outcomes measured titres of anti-AMA1 antibody (IgG) by ELISA and activity of anti-AMA1 antibody by growth inhibition assay (GIA). These assays were performed at baseline and at specified times during and after immunization (Additional file 1).

### Safety assessment

Following each immunization, participants were directly observed for 30 min, and then evaluated at the study clinic 1, 3, 7 and 14 days after each immunization and on study days 84, 140 and 364. Starting on day 140, monthly home visits were made to check the health status of participants and to encourage them to come to the research clinic if they felt ill. Clinical evaluations consisted of measurement of vital signs and assessment for local injection site and general solicited signs or symptoms. Local signs and solicited symptoms included pain, swelling, erythema at the injection site and limitation of arm abduction at the shoulder. General signs and solicited symptoms included fever (oral temperature ≥37.5 °C), chills, nausea, headache, malaise, myalgia and joint pain. Any other signs or symptoms were considered to be unsolicited, as were all signs or symptoms that occurred more than 7 days after immunization. All solicited local symptoms were considered related to the study vaccines. Pregnancies were monitored throughout the 12-month study period and until delivery, when this occurred after the study end. Blood was collected at screening, on immunization days and 7 days after each immunization and on study days 84, 140 and 364 to determine Complete blood count, biochemistry parameters (serum potassium, sodium, ASAT, ALAT, total bilirubin, alkaline phosphatase, γGT and creatinine). Adverse events were judged for relatedness to study vaccines and graded by severity as shown in Additional file 2: Table I. Laboratory parameters intensity was graded as indicated in Additional file 3: Table II.

### Immunogenicity assessment

Antibody responses to AMA-1 were measured by an enzyme-linked immunosorbent assay (ELISA). Briefly, IgG ELISAs were performed using PfAMA-1 FVO [25-545] from the same clinical batch which production was described by Faber et al. [9] and that was used in a Phase 1a trial in Nijmegen [11], as the capture antigen, in serial twofold dilution, and one arbitrary unit (AU) was defined at an optical density of 1.0 over background. Thus the titre in AU represents the dilution of the serum required to obtain an OD of 1 over background. Antibody responses were measured on serum obtained from participants at the time of each immunization (study days 0 [baseline]), 28, 56, 84, 140 and 364.

Pre-immunization (day 0) sera and sera from 4 weeks after the third immunization (day 84, corresponding to peak antibody titres) were tested for growth inhibitory effects against homologous parasites expressing AMA1 (derived from FVO clone) *P. falciparum* line as described [16, 17]. Antibodies used for growth inhibition assays were purified on protein G columns (Sigma, St Louis, MO) using standard protocols, exchanged into RPMI 1640 using Amicon Ultra-15 concentrators (30 kDa cutoff, Millipore, Ireland), filter-sterilized and stored at −20 °C until use. IgG concentrations were determined using a Nanodrop ND-1000 spectrophotometer (Nanodrop Technologies, Wilmington, DE, USA).

*Plasmodium falciparum* strain FCR3 was cultured in vitro using standard *P. falciparum* culture techniques in an atmosphere of 5 % CO_2_, 5 % O_2_ and 90 % N_2_. FCR3 AMA1 (accession no. M34553) differs by 1 amino acid in the pro-sequence from FVO AMA1 (accession no. AJ277646).

The effect of 5 or 10 mg mL^−1^ purified IgG antibodies on parasite invasion was evaluated in triplicate using 96 well flat-bottomed plates (Greiner) with in vitro matured and synchronized *P. falciparum* schizonts at a starting parasitaemia of 0.2–0.4 %, a haematocrit of 2.0 % and a final volume of 50 µL containing 10 % normal human serum, 20 µg mL^−1^ gentamicin in RPMI 1640. After 40 to 42 h, cultures were resuspended, and 50 µL was transferred into 200 µL ice-cold PBS. The cultures were then centrifuged, the supernatant removed and the plates were frozen. Inhibition of parasite growth was estimated using the pLDH assay as previously described [16]. Parasite growth inhibition, reported as a percentage, was calculated as follows: 100 − ((Od_experimental_ − Od _background_)/(Od_control_ − Od_background_) × 100). Control IgG was isolated from malaria naïve donors. The AMA1 expressed was verified to be FCR3 by RFLP analysis and parasite cultures used for GIA tested negative for Mycoplasma by PCR.

### Sample size

Determination of sample size was based on the primary safety endpoint, *i.e.* the occurrence of any solicited adverse events, unsolicited adverse events or change from baseline values of biological parameters during the study period. The primary analysis population was the intent-to-treat (ITT) population. Power calculations were based on the exact binomial distribution. With a sample size of 20 participants in each group, the study is powered to detect at least one adverse event or change from baseline values of biological parameters with a probability of at least 90 % if the incidence rate of adverse event or change from baseline values of biological parameters is 10.9 % or more. The study have a power of at least 80 % and 95 % to detect at least one adverse event or change from baseline values of biological parameters if their incidence rates are 7.7 % and 13.9 % or more, respectively. Incorporation of a comparator vaccine group of 20 permitted broad initial estimates of the incidence of local and general side effects and of immune responses among vaccine recipients.

### Randomization and masking

Individual participants were randomized in a 1:1 ratio to receive either PfAMA1-FVO/Alhydrogel^®^ or tetanus toxoid at the time of vaccination. Randomization to either of the two vaccines was done using a computer-generated randomization list. The randomization list contained sequential codes that linked a study number to a vaccine assignment. Study numbers were assigned to participants of each group in the order in which they were enrolled in the trial. The PfAMA1 vaccine and the comparator had different appearances. Therefore the vaccine preparation area was separated from the vaccine administration area. Vaccinations were carried out simultaneously in two separate consultation rooms, which were connected to a central pharmacy (the vaccine preparation room) by small windows with a sliding closure. On vaccination days, the prepared syringe was handed through the small window to a vaccinator for vaccine administration. In addition a study nurse not involved with assessment of study outcomes administered vaccines. And finally, the syringe containing vaccine was covered with opaque tape to mask its content and labelled with the participant study number and randomization code. At the study site, only the pharmacist, pharmacy assistant and the drug manager had access to the randomization list. Participants, investigators, lab personnel and all staff performing follow-up evaluations were blinded as to vaccine assignment. Following vaccine administration, participants were assessed and follow-up visits conducted by clinicians who had not been involved in the vaccinations.

### Data analysis

#### Data quality assurance

The trial was conducted in compliance with BMP Clinical Trial Quality Assurance Procedures and the MRTC Clinical Laboratory Quality Assurance Plan. The study sponsor ensured the external monitoring of the trial. Following the pre study assessment, study initiation, three routine monitoring and closeout visits were performed during the trial. Standard Case Report Forms pages monitored and validated were sent to MRTC Data Management Unit for data entry into a GCP compliant database. Data was double entered by two independent data entry clerks and then reconciled by the study statistician. After resolution of all discrepancies, the database was frozen and used for analysis.

#### Statistical methods

For all safety endpoints, adverse events (AEs) were summarized by grade and vaccine doses for each vaccination group. Categorical data are summarized using counts and percentages. For immunogenicity analysis, ELISA titres of AMA1 antibodies are plotted by vaccine group and time points together with the 95 % CI bars.

Comparisons of proportions between vaccine groups were done using Fisher exact test and Wilcoxon-Mann–Whitney test was used for comparison of distributions of ordinal or continuous data. Comparisons between baseline level and post-vaccination level of biological parameters were done using McNemar change test (for categorical data) or Wilcoxon signed ranks test (for ordinal or continuous data). Analysis of Mean Fold Increase (MFI) was done using a Pearson’s product moment correlation (r) and the slope and intercept were estimated using linear regression. The significance of the correlation coefficient was assessed by a *t* test. All tests were 2-sided, and no correction of p-values was made for additional analyses.

## Results

### Participant flow and baseline data

**Fig. 1:**
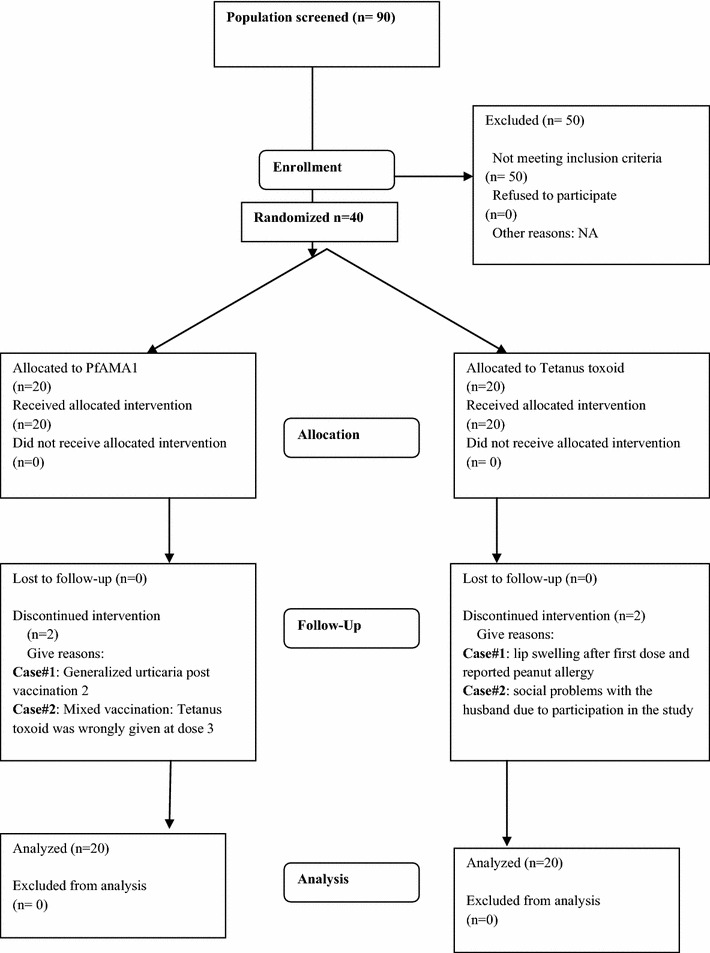
Trial profile

Ninety participants were screened, 40 were enrolled (26 females and 14 males) and all received the first dose of the vaccine according to study allocation (Fig. 1). Common reasons for exclusion were concurrent illnesses, laboratory abnormalities during screening. The mean age was 30.1 years in the malaria vaccine group and 26.0 in the control group. In the PfAMA1 group two participants were excluded from vaccination after the second dose; one because of urticaria deemed to be possibly related to vaccine due to time relationship and the second because of pruritis of unknown cause. In the tetanus toxoid group, one participant was excluded from vaccination after having developed a swelling of the upper lip post dose 1 and a history known only after inclusion, of allergy to peanuts. The two groups were generally similar at enrolment with regard to gender, age or laboratory parameters (Table 1).

**Table 1:** Baseline characteristics at enrolment

Characteristics	PfAMA1 N = 20	Tetanus toxoid N = 20	*p* value
Mean age in year ± SD	30.1 ± 10.6	26 ± 10.5	ns
Female (%)	70	65	ns
Mean WBC 10^3^/µL ± SD	6.0 ± 1.3	6.4 ± 1.4	ns
Mean RBC 10^3^/µL ± SD	4.6 ± 6	4.7 ± .4	ns
Mean Hemoglobin ± SD	13.3 ± 1.8	13.0 ± 1.2	ns
Mean Platelets10^3^/µL ± SD	245.2 ± 64.8	255.2 ± 76.0	ns
Mean Lymphocytes 10^3^/µL ± SD	2.6 ± 0.6	2.9 ± 0.8	ns
Mean Creatinin µM/l ± SD	62.7 ± 13.8	53.3 ± 5.5	ns
Mean ALT U/L ± SD	14.5 ± 5.5	12.7 ± 5.6	ns
GMT anti-AMA1 antibody titre (95 % CI)	5164 (2494–10,691)	9938 (4484–22,025)	ns

*GMT* geometric mean titre in arbitrary units (AU), *CI* confidence interval, *ALT* alanine Amino-transferase, *SD* standard deviation

### Safety and reactogenicity

Overall, the products showed a good safety profile similar to observations reported from previous trials. The 40 participants experienced a total of 257 adverse events, 136 were solicited AEs and 121 were unsolicited AEs. Additional vaccine doses did not globally increase the number of AEs.

### Local solicited adverse events

In the PfAMA1 group the most frequent local solicited AE was injection site pain reported at least by 60 % of the participants after any dose (Table 2) as compared to 40 % in the control group. All cases of injection site pain were of mild to moderate intensity and resolved within three days post-vaccination. Second in frequency was swelling at the injection site observed at least in 45 % of the participants after any of the vaccine dose. Injection site swelling was the only solicited AEs with grade 3 intensity. The grade 3 swelling was defined as a diameter superior to 50 mm and was not associated with pain or with other functional impairments. The incidence of local solicited AEs did not increase with subsequent vaccine dose administration. In the tetanus toxoid group, frequency of injection site pain and swelling was more balanced between participants, with most of events having a mild to moderate intensity (Table 2). One participant had grade 3 swelling that resolved within the three days of follow up.

**Table 2:** Signs and solicited symptoms during the 7-day follow-up periods after each immunization

Signs and solicited symptoms	PfAMA1 malaria vaccine						Tetanus toxoid vaccine					
	Dose 1 N = 20		Dose 2 N = 20		Dose 3 N = 20		Dose 1 N = 20		Dose 2 N = 20		Dose 3 N = 20	
	Overall	Severe	Overall	Severe	Overall	Severe	Overall	Severe	Overall	Severe	Overall	Severe
Local												
Pain (%)	12 (0.60)	0	9 (0.45)	0	6 (0.30)	0	8 (0.40)	0	2 (0.10)	0	2 (0.10)	0
Limited arm motion (%)	1 (0.05)	0	1 (0.05)	0	0	0	1 (0.05)	0	0	0	0	0
Swelling (%)	7 (0.35)	2 (0.10)	8 (0.40)	4 (0.20)	7 (0.35)	2 (0.10)	1 (0.05)	0	2 (0.10)	0	9 (0.45)	1 (0.05)
Erythema (%)	0	0	1 (0.05)	0	0	0	0	0	0	0	0	0
Systemic												
Fever (%)	2 (0.10)	0	0	0	0	0	1 (0.05)	0	3 (0.15)	0	1 (0.05)	0
Headaches (%)	5 (0.25)	0	6 (0.30)	0	3 (0.15)	0	10 (0.50)		4 (0.20)	0	1 (0.05)	0
Joint pain (%)	1 (0.05)	0	0	0	0	0	1 (0.05)	0	0	0	1 (0.05)	0
Malaise (%)	1 (0.05)	0	0	0	0	0	3 (0.15)	0	0	0	0	0
Myalgia (%)	2 (0.10)	0	4 (0.20)	0	1 (0.05)	0	2 (0.10)	0	0	0	0	0
Nausea (%)	3 (0.15)	0	0	0	0	0	1 (0.05)	0	0	0	0	0
Chills (%)	1 (0.05)	0	0	0	0	0	1 (0.05)	0	0	0	0	0

### Systemic solicited adverse events

All solicited systemic AEs were of minor (93.5 %) or moderate (6.5 %) intensity and consisted mainly of headache and myalgia. There was no appreciable difference between the two groups though the control group experienced double the number of headaches after dose 1.

### Unsolicited adverse events

Overall participants had a total of 123 unsolicited adverse events; 58 occurred in the malaria vaccine group and 65 in the control group. Unsolicited AEs classified by Body system and WHO preferred terms consisted mainly of respiratory disorders and infections, and were balanced by groups, being a representative picture of the morbidity usually observed in the study area.

One case of injection site induration of moderate intensity causally related to vaccine was reported in the malaria vaccine group after the second dose. The induration resolved within 4 weeks and the participant received the third vaccine dose. Three instances of pregnancy were reported, all after the study day 84. One case occurred in the control group and the participant decided to interrupt her pregnancy by abortion at the age of 4 weeks. Two others cases occurred in the malaria vaccine group; in both situations the pregnancy was diagnosed close to the last study clinic visit on study days 225 and 364. Both pregnancies resulted in healthy babies, one girl and one boy.

### Serious adverse event

One serious adverse event occurred in one participant in the tetanus toxoid group on study day 94. The diagnosis was an acute episode of food poisoning that resolved without sequelae. The event was not related to vaccination.

### Laboratory safety tests

Overall, the results showed a good biological safety profile. Only minor laboratory anomalies were reported and those consisted of grade 1 high Bilirubin levels in the malaria vaccine group after the first dose. These values returned to normal ranges before the second dose. There was no difference in mean haemoglobin levels between the two groups. No episode of anaemia defined as haemoglobin level ≤9.5 g/dL was observed among both vaccines recipients. The lowest haemoglobin level was 9.7 g/dL, recorded in a female participant on study days 35 and 84, in the malaria vaccine group.

### Immunogenicity

**Fig. 2:**
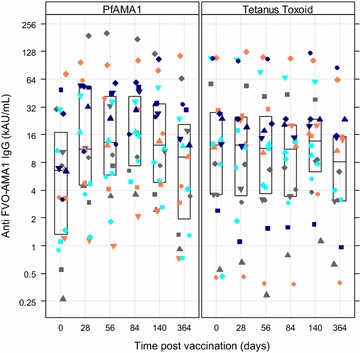
Anti FVO-AMA1 IgG titres. Individual data points are plotted as well as a box indicating median and 25 and 75 % quantiles. Same *symbol* and *color* refer to same individuals within treatment groups

**Fig. 3:**
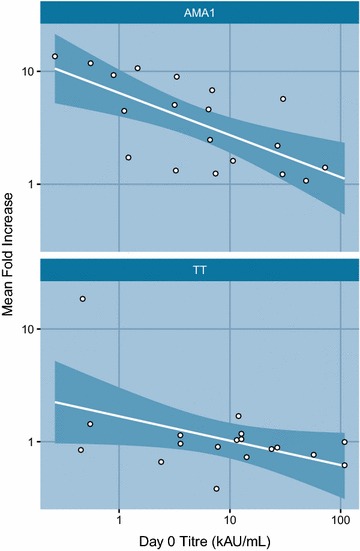
Mean fold Increase in IgG titres. Mean fold Increase (MFI = day84/day0) in IgG titres are plotted against day 0 IgG titres *Top panel* is PfAMA-1 group. *Bottom panel* is Tetanus Toxoid. *Dots* are fold increase. Regression line with 95 % CI. *AU* arbitrary units. kAU/mL = 1000 × AU/mL

IgG antibody levels to the FVO-AMA1 antigen are depicted in Figs. 2 and 3, and summary statistics are tabulated in Table 3. Baseline levels of anti-AMA1 antibodies were high in both groups and were higher in the tetanus toxoid group as compared to the PfAMA1 group; 5164, 95 % CI (2494 to 10,691) versus 9938, 95 % CI (4484 to 22,025), respectively. The PfAMA1 vaccine induced a significant increase in AMA1-specific IgG (*i.e.*, ratio day x/baseline >1) at all time points following vaccination (p < 0.05) (Table 3); after vaccination, titres increased gradually in the PfAMA1 recipients until day 84 when a maximum level was observed with a geometric mean of 17,584 arbitrary units 95 % CI (9889 to 31,267). The PfAMA1 group showed a 3.5, 95 % CI (2.3–5.4) fold rise in the IgG titres by day 84 as compared to baseline levels, while in the control group the day 84 fold rise compared to baseline was 1.07, 95 % CI (0.7–1.6). Thereafter, IgG titres decreased gradually during the study. This group assessment covers a wide range of individual variability. In the PfAMA1 group the highest increase in antibody titres was 60-fold increase reported after second vaccination. Those with the highest increase had the lowest baseline levels. In the PfAMA-1 group, MFI was significantly and negatively correlated with day 0 IgG titre (*p* = 0.002). The correlation coefficient was −0.67 with 95 % CI (−0.86 to −0.31). In the malaria vaccine group antibody responses were not sustained and returned to baseline levels after 12 months.

**Table 3:** IgG Titres to the immunising antigen at specified time points

Day	PfAMA1		Tetanus toxoid	
	IgG (95 % CI)	N	IgG (95 % CI)	N
Panel A. IgG titres to AMA1 FVO				
0	5164 (2494–10,691)	20	9938 (4484–22,025)	20
28	13,888 (7017–27,484)	20	10,475 (4892–22,428)	20
56	14,954 (7923–28,222)	20	9192 (4103–20,591)	19
84	17,584 (9889–31,267)	19	8744 (4184–18,276)	18
140	14,048 (7808–25,275)	20	12,793 (7187–22,773)	20
364	6925 (3295–14,555)	19	6551 (2896–14,818)	16
Panel B. Titre changes following vaccination (fold increase)				
0	1	20	1	20
28	2.69 (1.73–4.18)	20	1.05 (0.87–1.28)	20
56	2.90 (1.87–4.48)	20	0.93 (0.70–1.25)	19
84	3.53 (2.30–5.42)	19	1.07 (0.72–1.58)	18
140	2.72 (1.88–3.94)	20	1.29 (0.86–1.93)	20
364	1.46 (1.02–2.09)	19	0.89 (0.61–1.28)	16

### Growth inhibition assays

GIA titres are summarized in Table 4. Baseline levels of GIA were high in both groups: PfAMA1 and control vaccines recipients had baseline GIA levels of 56 (25–44) and 59 (19–50), respectively. Vaccination did not change GIA titres resulting in day 84 GIA titres of 68 (7–64) and 62 (19–53) for the PfAMA1 and control group, respectively. GIA titres decreased slightly by day 365, with GIA titres of 43 (26–31) and 49 (25–36) for PfAMA1 and controls, respectively.

**Table 4:** GIA titres at 10 mg/mL IgG in vaccinated and control volunteers

Day	PfAMA1 Malaria Vaccine		Tetanus toxoid vaccine	
	N	GIA (95 % CI)	N	GIA (95 % CI)
0	20	56 (25–44)	20	59 (19–50)
84	19	68 (7–64)	18	62 (19–53)
365	19	43 (26–31)	17	49 (25–36)

## Discussion

This study is the first evaluation of the AMA1-based malaria vaccine PfAMA1-FVO [25-545] adjuvanted with alum in malaria-experienced adults. The vaccine showed acceptable tolerability. Local reactions were more frequent in malaria vaccine groups than in the comparator group. Most recipients of the malaria vaccine experienced pain at the injection site. The incidence of injection site pain did not increase with subsequent administration of doses of the vaccine. Although swelling was often classified and graded based on the size of the reaction, these episodes of swelling were short-lived and were not associated with pain or with other functional impairments. There was a slight, non-statistical significant tendency to more adverse events in the control group than in the PfAMA1 group.

The safety and tolerability profile of the PfAMA1-FVO [25-545]/Alhydrogel^®^ vaccine was similar to that seen in a previous trial of this vaccine in malaria-naïve volunteers [9]. Others formulations of AMA1 using different adjuvant system also showed acceptable tolerance profile in malaria-naïve [18–20] and Malian adults [3, 4, 21]. Biological parameters assessed have remained within area normal ranges. Anaemia was a concern in AMA1-based malaria vaccines tested in children [5, 22]. In this adult population, no anaemia cases were observed. A female participant recipient of the malaria vaccine had decreased levels of haemoglobin measured at two time points. However, these decreased levels were above the threshold that defined anaemia in the study population.

Antibody titres peaked 1 month after the third dose reaching a 3.5 fold rise. A comparable dynamic in antibody titres was reported in naive volunteers [9]. There is a very clear relation between pre-immunization titre and fold rise. The highest pre-titre have resulted in the lowest fold rise. In this trial, immunization started with the rainy season when most malaria transmission occurs in the study area. However high antibody titres were not sustained and returned to baseline levels after 12 months. This could witness an absence of natural boosting. Previous trials of AMA1 using AS02, a more potent adjuvant system, have generated much higher antibody titres [4], that persisted in children more than 12 months [23].

No significant change in GIA activities were observed, despite the rise in IgG titres. In previous trials, serum from naive adults immunized with AMA-1 3D7 did not show good grow inhibition on heterologous (FVO) parasites as compared to homologous (3D7) parasites [24]. Interestingly the immune response elicited by AMA1 from *P. falciparum* 3D7 in semi-immune adults showed a higher grow inhibitory activity on FVO clone than 3D7 clone of *P. falciparum* [4].

The functionality of immune response induced by AMA-1 may not be function of the system of expression of recombinant protein used, since the AMA-1 FVO expressed in *Pichia pastoris* and *Escherichia coli* has shown similar functionality in growth inhibitory experiments [25]. The adjuvant system might play a more prominent role in inducing functional antibodies.

Apical Membrane Antigen 1 is an extremely polymorphic protein, with more than 100 polymorphic amino acid sites, and in vitro experiments and studies in both animals and humans have indicated some degree of allele-specificity in the antibody responses to genetically different forms of AMA-1 [26, 27]. The PfAMA1 vaccine is based on AMA-1 sequence from the FVO clone of *P. falciparum* and other AMA-1-based vaccines developed from both 3D7 and FVO [4, 10, 19, 21, 24] have given good immune responses and acceptable reactogenicity. A phase 2b trial conducted in the same site assessed the efficacy of the 3D7 derived AMA-1 associated with AS02A did not have significant efficacy against clinical malaria episodes but showed high allele-specific efficacy against clinical malaria [28]. Vaccine allele-specific efficacy depended on the degree of homology at key amino acid residues between the vaccine antigen and AMA-1 in parasites circulating at the vaccine trial site [29, 30].

The PfAMA1-FVO [25-545] malaria vaccine candidate clinical development was stopped after the present trial was completed, partly because of the potential limits imposed by strain specificity of protection to polymorphic AMA1 confirmed in human [28]. However efforts were pursued to overcome strain-specificity of protection. The diversity covering approach developed a couple of years before by the same team at the Biomedical Primate Research Centre [31–33] that developed the PfAMA1-FVO [25-545], is promising and have now gone into clinical trials in malaria endemic settings [34].

## Conclusion

The PfAMA1-FVO [25-545] malaria vaccine candidate was safe and well tolerated in adult exposed to intense and seasonal malaria transmission in Mali. The vaccine induced high antibody response that was not however sustained over the malaria transmission season.

## Additional files

10.1186/s12936-016-1466-4 Raw data set of the IgG and GIA responses for the phase 1 trial of PfAMA1 FVO [25-545] in Malians adults in Bandiagara.
10.1186/s12936-016-1466-4 Solicited adverse event intensity scale.
10.1186/s12936-016-1466-4 Abnormal laboratory parameters grading intensity.

## Abbreviations

ALT (ALAT): Alanine transaminase (Alanine amino transferase)
AST (ASAT): Aspartate transaminase (Aspartate amino transferase)
BMP: Bandiagara Malaria Project
°C: Degree Celsius or centigrade
CI: Confidence interval
CO_2_: Carbon dioxide
dL: Decilitre
EDTA: Ethylene diamine tetraacetic acid
ELISA: Enzyme-linked immunosorbent assay
FMPOS: Faculty of Medicine, Pharmacy and Odonto-Stomatology of Bamako, Mali
FVO, FCR3: *Plasmodium falciparum* parasites clones
GCP: Good clinical practice
GIA: Growth inhibition assay
GMP: Good manufacturing practice
γGT: Gamma-glutamyl transferase
IgG: Immunoglobulin G
kDa: KiloDaltons
mg: Milligramme
mL: Millilitre
MRTC: Malaria Research and Training Center
OD: Optical density
O_2_: Oxygen
PBS: Phosphate-buffered saline
PCR: Polymerase chain reaction
PfAMA1[25-545]: *Plasmodium falciparum* Apical membrane Antigen 1, amino acids 25–545
pLDH: *Plasmodium falciparum* lactate dehydrogenase
RFLP: Restriction fragment length polymorphism
RPMI 1640: Roswell Park Memorial Institute culture medium
SA: Société Anonyme
µg: Microgramme
WHO: World Health Organization
