# Supplementary material for: Phase 1 randomized controlled trial to evaluate the safety and immunogenicity of recombinant Pichia pastoris-expressed Plasmodium falciparum apical membrane antigen 1 (PfAMA1-FVO [25-545]) in healthy Malian adults in Bandiagara
Source: Malar J. 2016 Aug 30;15(1):442. doi: 10.1186/s12936-016-1466-4 (PMC5006270; doi:10.1186/s12936-016-1466-4)
Supplement: Supplementary file 2 — 10.1186/s12936-016-1466-4 Solicited adverse event intensity scale. [file 12936_2016_1466_MOESM2_ESM.docx]

Table I. Solicited adverse event intensity scale

| **Adverse Event** | **Intensity grade** | **Intensity definition** |
| --- | --- | --- |
| Pain at injection site | 0 | None |
|  | 1 | Pain that is easily tolerated |
|  | 2 | Pain that interferes with daily activity |
|  | 3 | Pain that prevents daily activity |
| Swelling at injection site  Record size | 0  1  2  3 | 0 mm  >0 - ≤ 20 mm  >20 - ≤ 50 mm  >50 mm |
| Erythema at injection site  Record size | 0  1  2  3 | 0 mm  >0 - ≤ 20 mm  >20 - ≤ 50 mm  >50 mm |
| Limitation of arm motion - | 0 | None |
| Abduction at the shoulder | 1 | >90° but <120° |
|  | 2 | >30° but ≤90° |
|  | 3 | ≤30° |
| Fever  Record oral temperature | 0  1  2  3 | <37.5ºC  37.5 - ≤38.0ºC  >38.0 - ≤39ºC  >39ºC |
| Chills | 0 | None |
|  | 1 | Chills that are easily tolerated |
|  | 2 | Chills that interfere with daily activity |
|  | 3 | Chills that prevent daily activity |
| Nausea | 0 | None |
|  | 1 | Nausea that is easily tolerated |
|  | 2 | Nausea that interferes with daily activity |
|  | 3 | Nausea that prevents daily activity |
| Headache | 0 | None |
|  | 1 | Headache that is easily tolerated |
|  | 2 | Headache that interferes with daily activity |
|  | 3 | Headache that prevents daily activity |
| Malaise | 0 | None |
|  | 1 | Malaise that is easily tolerated |
|  | 2 | Malaise that interferes with daily activity |
|  | 3 | Malaise that prevents daily activity |
| Myalgia | 0 | None |
|  | 1 | Myalgia that is easily tolerated |
|  | 2 | Myalgia that interferes with daily activity |
|  | 3 | Myalgia that prevents daily activity |
