# Supplementary material for: Phase 1 randomized controlled trial to evaluate the safety and immunogenicity of recombinant Pichia pastoris-expressed Plasmodium falciparum apical membrane antigen 1 (PfAMA1-FVO [25-545]) in healthy Malian adults in Bandiagara
Source: Malar J. 2016 Aug 30;15(1):442. doi: 10.1186/s12936-016-1466-4 (PMC5006270; doi:10.1186/s12936-016-1466-4)
Supplement: Supplementary file 3 — 10.1186/s12936-016-1466-4 Abnormal laboratory parameters grading intensity. [file 12936_2016_1466_MOESM3_ESM.docx]

Table II: Abnormal laboratory parameters grading intensity:

| **Grade** | **1** | **2** | **3** | **4** |
| --- | --- | --- | --- | --- |
| Hemoglobin (g/dL) |  |  |  |  |
| Male | 8.0 - 10.0 | 6.6 - 7.9 | 5.0 - 6.5 | < 5.0 |
| Female | 8.5 - 9.5 | 7.5 - 8.4 | 6.5 - 7.4 | < 6.5 |
| Absolute Lymphocyte Count (cells/mm^3^) | 1000-1500 | 750-999 | 500-749 | <500 |
| Platelets (cells/mm^3^) | 75000-99000 | 50000-74999 | 20000-49000 | <20000/mm^3^ |
| WBCs (cells/mm^3^) | 11,500 – 13,000  or  2,000 – 3,000 | 13,000 –15,000  or  1,500 – 2,000 | 15,000 – 30,000  or  1,000 – 1,500 | >30,000  or  <1,000 |
| Creatinine (µmol/l) |  |  |  |  |
| Male | 111-152 | 153-303 | 304- 606 | > 606 or  dialysis required |
| Female | 71.8-100 | 101-200 | 201-392 | >392 |
| ALT (UI/l) | 54 – 108 | 109-216 | 217-433 | > 433 |
